# E3 Ligases Regulate Organelle Inheritance in Yeast

**DOI:** 10.3390/cells13040292

**Published:** 2024-02-06

**Authors:** Keisuke Obara, Kohei Nishimura, Takumi Kamura

**Affiliations:** Department of Biological Science, Graduate School of Science, Nagoya University, Furo-Cho, Chikusa-Ku, Nagoya 464-8602, Japan; nishimura.kohei.x8@f.mail.nagoya-u.ac.jp

**Keywords:** ubiquitin, E3 ligase, proteolysis, vacuoles, mitochondria, peroxisomes, myosin, yeast

## Abstract

*Saccharomyces cerevisiae* proliferates by budding, which includes the formation of a cytoplasmic protrusion called the ‘bud’, into which DNA, RNA, proteins, organelles, and other materials are transported. The transport of organelles into the growing bud must be strictly regulated for the proper inheritance of organelles by daughter cells. In yeast, the RING-type E3 ubiquitin ligases, Dma1 and Dma2, are involved in the proper inheritance of mitochondria, vacuoles, and presumably peroxisomes. These organelles are transported along actin filaments toward the tip of the growing bud by the myosin motor protein, Myo2. During organelle transport, organelle-specific adaptor proteins, namely Mmr1, Vac17, and Inp2 for mitochondria, vacuoles, and peroxisomes, respectively, bridge the organelles and myosin. After reaching the bud, the adaptor proteins are ubiquitinated by the E3 ubiquitin ligases and degraded by the proteasome. Targeted degradation of the adaptor proteins is necessary to unload vacuoles, mitochondria, and peroxisomes from the actin–myosin machinery. Impairment of the ubiquitination of adaptor proteins results in the failure of organelle release from myosin, which, in turn, leads to abnormal dynamics, morphology, and function of the inherited organelles, indicating the significance of proper organelle unloading from myosin. Herein, we summarize the role and regulation of E3 ubiquitin ligases during organelle inheritance in yeast.

## 1. Introduction

During cell proliferation, organelles are inherited by newly generated cells. The budding yeast *Saccharomyces cerevisiae* proliferates by budding, a process characterized by the formation of a cytoplasmic protrusion called the ‘bud’, which grows as the cell cycle progresses and eventually separates to become an independent daughter cell. Proliferation by budding involves the bud-directed transport of DNA, RNA, proteins, organelles, and other materials that are essential for daughter cells. The directed transport of organelles toward the growing bud is mainly mediated by class V myosins, Myo2 and Myo4, on actin filaments [1,2]. Cortical ER is carried by Myo4, whereas Myo2 transports most other organelles such as the Golgi apparatus, vacuoles, peroxisomes, and mitochondria [3,4,5,6,7,8,9,10,11]. At the late stage of budding, namely the cytokinesis stage, Myo2 translocates from the bud tip to the bud neck to deliver vesicles containing proteins and other materials required for the formation of the septum that physically separates the mother and daughter cells. Therefore, the transported organelles should be released from Myo2 once they arrive at their destination, which is inside the growing bud, to avoid backward flow to the bud neck and spatial hindrance to cytokinesis progression. For vacuoles, mitochondria, and presumably peroxisomes, unloading from the actin–myosin machinery after delivery to the bud requires the ubiquitination and degradation of adaptor proteins [12,13]. The proteolysis of the adaptor proteins is spatiotemporally regulated mainly by their phosphorylation. This review focuses on the mechanism and significance of the unloading of vacuoles, mitochondria, and peroxisomes from the actin–myosin machinery during their inheritance in yeast.

## 2. Vacuole Inheritance and E3 Ligases

The involvement of ubiquitin ligases in organelle inheritance in yeast was first discovered and has since been extensively studied in vacuoles. Prior to directed transport to the bud, vacuoles fragment and/or tubulate to form ‘segregation structures’ that extend from the vacuoles in the mother cell [14]. The segregation structure is transported into the growing bud by Myo2 along actin filaments. Myo2 captures vacuoles through the vacuole-specific adaptor protein Vac17 [5,6]. The interaction between Myo2 and Vac17 is coordinated by cell cycle progression and is directly regulated by the phosphorylation of Vac17 by the cyclin-dependent kinase Cdk1/Cdc28 [15]. Proper vacuole inheritance also requires the protein phosphatase Ptc1 [16]. Ptc1 is required to maintain Vac17 levels and has been proposed to play a role in the formation of a transport complex containing Myo2 and Vac17. Interestingly, Ptc1 has also been proposed to regulate the formation of transport machinery for other organelles, such as mitochondria and peroxisomes (see Discussion and Future Perspectives). Vac17 binds to the vacuole via Vac8, which attaches to the vacuolar membrane via palmitoylation [17,18]. After vacuoles enter the growing bud, they are released from the actin–myosin machinery.

**Figure 1:**
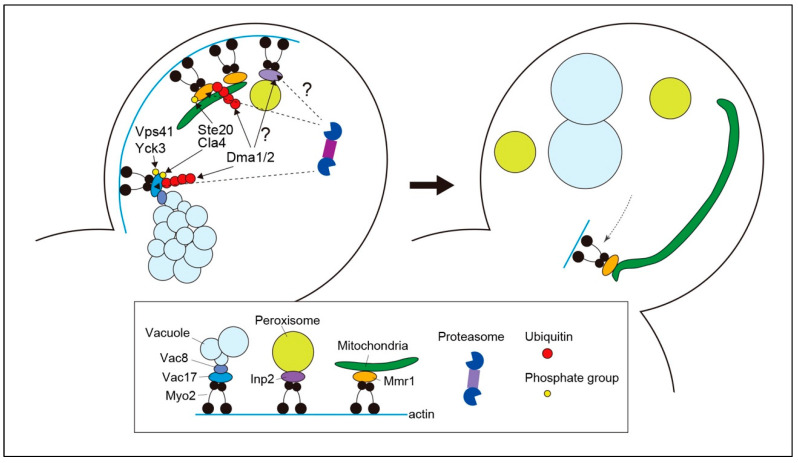
Organelle unloading from myosin during budding in yeast. Myo2 captures vacuoles, mitochondria, and peroxisomes in mother cells through organelle-specific adaptors, such as Vac17, Mmr1, and Inp2. The vacuolar protein Vac8 is involved in bridging vacuoles and Vac17. As the vacuole enters the growing bud, Vac17 undergoes phosphorylation, which is required for its unloading from myosin. A yet-to-be-identified kinase phosphorylates Vac17 to recruit E3 ubiquitin ligases Dma1 and Dma2. Phosphorylation of Vac17 at Ser222 residue by Cla4 and Ste20 triggers Vac17 ubiquitination by Dma1 and Dma2. Because Cla4 and Ste20 are mostly confined to the bud cortex, Cal4- and Ste20-mediated phosphorylation may serve as a spatial cue for initiating the degradation processes. Parallel to this axis, Vac17 is phosphorylated depending on Yck3 and Vps41, which are thought to be required for the dissociation of Vac17 from Myo2, thereby facilitating access of the Cdc48 complex which guides the ubiquitinated proteins to the proteasome. Both ubiquitination by Dma1 and Dma2 and phosphorylation dependent on Yck3 and Vps41 are required for efficient Vac17 degradation and vacuole release from the actin–myosin machinery. During mitochondrial unloading, Mmr1 is phosphorylated at S414 residue by Cla4 and Ste20, which is a prerequisite for its ubiquitination by Dma1 and Dma2. Ubiquitinated Mmr1 is degraded by the proteasome, resulting in the release of mitochondria from Myo2. A minor fraction of Mmr1, typically around one end of the tubular mitochondria, appears to escape degradation and maintain its association with Myo2. As Myo2 translocates to the bud neck during cytokinesis, the ends of the mitochondria with residual Mmr1 are targeted to the bud neck and anchored. Although Dma1 and Dma2 are involved in normal peroxisome dynamics in buds, the precise mechanism underlying their unloading from Myo2 remains unknown.

The release of vacuoles from the actin–myosin machinery in the bud is mediated by the proteolysis of Vac17 by the ubiquitin–proteasome system [13] (Figure 1). Vac17 is a short-lived protein containing a PEST motif that often acts as a signal for protein degradation [6,19]. The PEST motif of Vac17 is required for rapid Vac17 degradation and the release of vacuoles from the actin–myosin machinery. E3 ubiquitin ligase Dma1 is required for Vac17 degradation and vacuole detachment from Myo2 [13]. Dma1 is a RING-type E3 ubiquitin ligase. Dma1 and its paralog Dma2 have been implicated in the regulation of septin dynamics and the spindle position checkpoint [20]. These findings shed light on a novel and unexpected role of Dma1/2 in organelle inheritance. In *dma1*Δ cells, Vac17 levels are elevated, and vacuoles are mistargeted to the bud neck, where Myo2 translocates during cytokinesis [13]. Dma1 and Dma2 are involved in Vac17 ubiquitination in vivo, and Dma1 plays a major role in vacuole release compared to Dma2. Therefore, vacuole release from the actin–myosin machinery requires ubiquitination of Vac17 by Dma1 and Dma2 and its subsequent proteolysis.

The degradation of Vac17 must be regulated spatially and temporally to avoid the unloading of the vacuole before it reaches its destination, that is, the growing bud. Vac17 phosphorylation and dephosphorylation during vacuole inheritance play critical roles in the spatiotemporal regulation of Vac17 attachment to Myo2, its ubiquitination, and its detachment from Myo2. During the initial steps of vacuole inheritance, Cdk1/Cdc28 phosphorylates Vac17 to mediate Vac17-Myo2 attachment [15]. The dephosphorylation of Vac17 by Ptc1 is also an important regulatory step in the initiation of vacuole transport [16]. The next regulatory step is the phosphorylation of Vac17 at Thr240 in the PEST motif by a yet-to-be-identified kinase, which is required for recruiting Dma1 to Vac17. Interestingly, the recruitment of Dma1 alone is not sufficient for Vac17 ubiquitination. Recruited Dma1 is activated by the additional phosphorylation of Vac17 at Ser222 by Cla4, a member of the p21-activated kinase family [21]. Since Cla4 localizes to the bud cortex and is nearly absent from the mother cell, Cla4-dependent phosphorylation of the Vac17 Ser222 residue is thought to be a spatial cue that ensures Vac17 ubiquitination only after the vacuole has entered the growing bud [21]. The mechanism by which phosphorylation of the Vac17 Ser222 residue activates Dma1 remains unknown. Ubiquitination of Vac17 by Dma1 and Dma2 is insufficient for Vac17 degradation and vacuole release from the actin–myosin machinery. Vac17 degradation requires additional phosphorylation of Vac17, which involves Yck3 and Vps41 [22]. In *yck3*Δ and *vps41*Δ cells, ubiquitinated Vac17 accumulates, and vacuoles are mistargeted to the bud neck during cytokinesis. Yck3- and Vps41-dependent Vac17 phosphorylation has been proposed to facilitate the dissociation of Vac17 from Myo2, which may be required for the efficient exposure of ubiquitinated Vac17 to the Cdc48 complex, which guides ubiquitinated substrates to the proteasome [23]. Although Vps41 is a subunit of the homotypic fusion and vacuole protein sorting (HOPS) tethering complex involved in vacuole fusion [24,25], its role in vacuole release from the actin–myosin machinery is independent of the HOPS complex [22].

## 3. Mitochondria Inheritance and E3 Ligases

Mitochondria are transported to the growing bud by Myo2 along actin filaments for inheritance by daughter cells [9,10]. Myo2 captures mitochondria via the adaptor protein Mmr1 [26]. Mmr1 binds to the mitochondria by interacting with lipid molecules on the outer membrane via its basic amino acid residues [27]. After being transported to the growing bud, the mitochondria are released from the actin–myosin machinery and move dynamically in the daughter cells. The mitochondria are mainly inherited by daughter cells via this pathway. Ypt11 is a small G protein involved in a parallel pathway for the inheritance of mitochondria to the daughter cells [9,10]. Cells lacking both *MMR1* and *YPT11* are lethal, whereas each single-deletion mutant is viable.

In budding yeast, daughter cells preferentially receive healthy mitochondria, that is, mitochondria with greater reducing power and lower levels of reactive oxygen species (ROS) generation [28]. Yeast cells are also equipped with a system to preserve a portion of healthy mitochondria in the mother cell to avoid the complete loss of high-quality mitochondria. The retention of healthy mitochondria is mediated by their anchoring to mother cells via Mfb1 [29,30].

**Figure 2:**
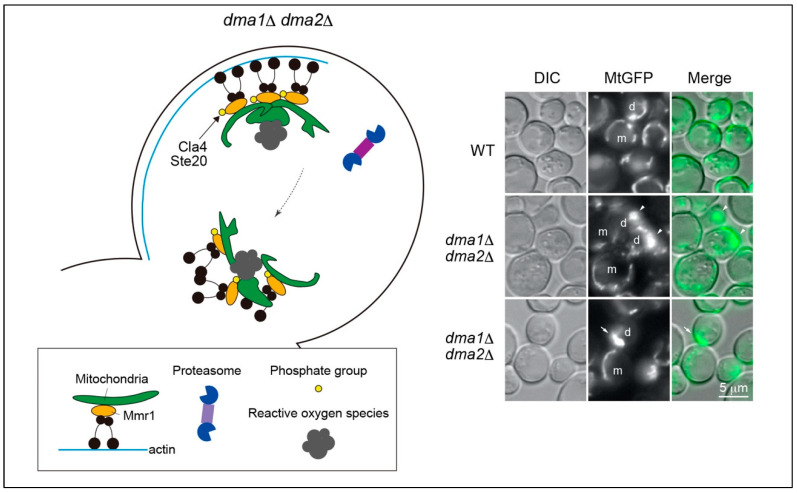
Abnormal mitochondrial dynamics and morphology in *dma1*Δ *dma2*Δ cells. In *dma1*Δ *dma2*Δ cells, Mmr1 fails to undergo ubiquitination and is not degraded, even after the mitochondria reach the bud tip. Consequently, the mitochondria are stacked at the bud tip, entwined, and deformed into abnormal shapes. However, the mechanism through which mitochondrial stacking leads to deformation remains unclear. During cytokinesis, mitochondria exhibit backward movement toward the bud neck because of Myo2 translocation. Stacked mitochondria have abnormally elevated respiratory activity and accordingly generate higher levels of reactive oxygen species than normal mitochondria in WT cells. The right panel shows fluorescence micrographs of mitochondria. The mitochondria were visualized using GFP fused to the mitochondrial pre-sequence of F_0_-ATPase subunit 9 (MtGFP). Cells in logarithmic growth phase were microscopically analyzed. Mitochondria were stacked at the bud tip (arrowheads) or neck (arrow) in *dma1*Δ *dma2*Δ cells. m, mother cell; d, daughter cell; DIC, differential interference contrast microscopy.

The ubiquitin–proteasome system has recently been reported to be involved in mitochondrial inheritance [12]. The adaptor protein, Mmr1, is a short-lived protein [31] that is rapidly degraded by the proteasome (Figure 1). The redundant E3 ligases, Dma1 and Dma2, were detected in the fraction of Mmr1-interacting proteins and were shown to ubiquitinate Mmr1 both in vivo and in vitro. Defects in Mmr1 ubiquitination in *dma1*Δ *dma2*Δ cells lead to a failure to unload mitochondria from the actin–myosin machinery in the bud. Accordingly, mitochondria in *dma1*Δ *dma2*Δ cells are first stacked at the bud tip, then exhibit backward movement from the bud tip to the bud neck, together with Myo2, and become stacked again during cytokinesis (Figure 2). Sometimes, mitochondria that move back to the bud neck are sorted into the mother cell after septum formation is completed, resulting in excess mitochondria in the mother cell and, conversely, a shortage in the daughter cell. Mitochondria stacked at the bud tip and neck in *dma1*Δ *dma2*Δ cells are intricately entwined and expanded or deformed into an abnormal shape (see Discussion and Future Perspectives). The regulation of respiratory activity seems to be deficient in these deformed mitochondria in *dma1*Δ *dma2*Δ cells [12]. When grown in a medium containing glucose as the carbon source, yeast cells activate the glycolytic pathway for energy production. Conversely, aerobic respiration is maintained at a low level. When carbon sources are non-fermentable, e.g., glycerol, yeast cells activate aerobic respiration for energy production. Therefore, defects in aerobic respiration lead to the lethality or slow-growth phenotype of yeast cells in a medium containing only non-fermentable carbon sources. Interestingly, *dma1*Δ *dma2*Δ cells grow faster than WT cells on a plate containing glycerol as the sole carbon source, although they grow slightly slower than WT cells on glucose-containing medium, which suggests an elevation of respiratory activity in *dma1*Δ *dma2*Δ cells [12]. Indeed, mitochondrial membrane potential, which often reflects respiratory activity, is abnormally high in deformed mitochondria in *dma1*Δ *dma2*Δ cells, and levels of cytochrome *c*, an electron carrier in the electron transport chain, are higher in *dma1*Δ *dma2*Δ cells than in WT cells in both glucose- and glycerol-containing media. The dysregulation of respiratory activity in stacked mitochondria in *dma1*Δ *dma2*Δ cells is accompanied by the generation of higher levels of ROS compared to that generated in normal mitochondria in WT cells. As a result, *dma1*Δ *dma2*Δ cells are hypersensitive to oxidative stress caused by the ROS-generating reagent paraquat or by the deletion of genes encoding the superoxide dismutases *SOD1* and *SOD2*. These phenotypes, derived from the dysregulation of respiratory activity in *dma1*Δ *dma2*Δ cells, can be interpreted as follows: in glucose-containing media, the negative effect of elevated ROS generation may overwhelm the positive impact of effective ATP synthesis by increasing respiratory activity. In contrast, in a medium containing glycerol as the sole carbon source, the benefit of elevated respiratory activity surpasses the toxicity of ROS production because ATP synthesis depends on respiration in this medium [12]. In summary, the ubiquitination and degradation of Mmr1 are essential for the normal dynamics, morphology, and function of mitochondria.

Similar to vacuole inheritance, the release of mitochondria from the actin–myosin machinery is spatially and temporally regulated by phosphorylation [12,21]. Bud-localized kinases Cla4 and Ste20 phosphorylate Ser414 of Mmr1. Phosphorylation at Ser414 is a prerequisite for Mmr1 ubiquitination by Dma1 and Dma2. The Mmr1-S414A mutant protein has a prolonged lifetime compared to that of the WT Mmr1 protein, and cells expressing this mutant Mmr1 exhibit mitochondrial stacking at the bud tip and neck, similar to *dma1*Δ *dma2*Δ cells. Restricted localization of Cla4 and Ste20 to the bud cortex may guarantee the unloading of mitochondria from the actin–myosin machinery only after the mitochondria enter the growing bud. As mentioned above, the degradation of the vacuolar-specific adaptor Vac17 requires Yck3- and Vps41-dependent phosphorylation. In contrast, Yck3 and Vps41 are dispensable for rapid degradation of Mmr1. Whether additional phosphorylation of Mmr1 by a pathway other than the Yck3–Vps41 axis is required for the release of mitochondria from the actin–myosin machinery remains unknown and is an important topic for future consideration.

Interestingly, a minor fraction of Mmr1 on the mitochondria, particularly around one of the two ends of the tubular mitochondria, appears to escape degradation and maintain its association with Myo2 during cytokinesis [12]. Residual Mmr1 is observed at the bud neck, probably because of the translocation of Myo2 to the bud neck. Consequently, one end of the tubular mitochondria is anchored to the bud neck and acts as a fulcrum that supports the dynamic movement of other parts of the mitochondria in the daughter cells (Figure 1). The mechanism and biological function of the selective protection of Mmr1 from degradation are unknown and will lead to interesting future studies.

## 4. Peroxisome Inheritance and E3 Ligases

Bud-directed transport of peroxisomes is also mediated by Myo2 along actin filaments. Similar to vacuoles and mitochondria, an organelle-specific adaptor protein, Inp2, bridges Myo2 to the peroxisomes [8,32]. Inp2 is a peroxisome membrane protein that contains a PEST motif and several substrate-recognition sites for Cdk1/Cdc28 [8,33]. A fraction of the peroxisomes are anchored to the mother cell to maintain the balance of the peroxisome population between the mother and daughter cells. The retention of peroxisomes in mother cells is mediated by Inp1, which bridges Pex3 on both the peroxisome and mother cortical ER membranes [33,34]. The overexpression of Inp1 results in the loss of peroxisomes in growing buds [35]. Inp1 is also implicated in the anchoring of peroxisomes to the bud after transport; loss of Inp1 causes the backward translocation of peroxisomes to mother cells [35].

E3 ubiquitin ligases may be involved in unloading peroxisomes from the actin–myosin machinery after they travel to the growing bud. In *dma1*Δ *dma2*Δ cells, peroxisomes are stacked at the bud neck during cytokinesis, similar to vacuoles and mitochondria [13], implying that the same scheme as the unloading of vacuoles and mitochondria can be applied to peroxisomes. The mechanisms of recruitment of Dma1 and Dma2 to substrates on the peroxisome and their activation remain unknown.

## 5. Discussion and Future Perspectives

The unloading of vacuoles and the unloading of mitochondria largely share the same mechanism (Figure 1). First, degradation of the adaptor proteins Vac17 and Mmr1 for vacuoles and mitochondria, respectively, is required for organelle release from the actin–myosin machinery. Second, the same E3 ubiquitin ligases, Dma1 and Dma2, are involved in the ubiquitination of adaptor proteins. Third, the phosphorylation of adaptor proteins serves as a spatial cue to trigger their ubiquitination for adaptor proteins to be degraded only once the cargo has reached its destination, that is, the growing bud. Fourth, kinases that lead to the ubiquitination of adaptors, such as the p21-activated kinases Cla4 and Ste20, are shared during the unloading of vacuoles and mitochondria. Although the mechanism of peroxisome release from Myo2 remains largely unknown, it is noteworthy that peroxisomes are stacked at the bud neck in *dma1*Δ *dma2*Δ cells, like vacuoles and mitochondria. Overall, it is likely that a common strategy in yeast cells is to degrade adaptor proteins using the ubiquitin–proteasome system to unload inherited organelles.

Employing similar transport and unloading mechanisms for different organelles appears to be an efficient method of controlling organelle inheritance in yeast cells. However, at the same time, such a simple system may also require an additional tight and elaborate coordination system to avoid competition for transport/unloading machinery or entanglement of inheritance pathways among different organelles. Although different organelles are carried by the same motor protein, Myo2, in yeast, they are eventually localized to distinct positions in the bud at different times during budding [36,37], which implies the existence of systems to spatiotemporally coordinate the Myo2-dependent inheritance of distinct organelles. One such regulatory mechanism may involve controlling the levels of adaptor proteins via cell-cycle-coupled regulation of gene expression and protein turnover [8]. As extensively studied in vacuole inheritance, phosphorylation and dephosphorylation of adaptor proteins are also parts of such coordination mechanisms. As mentioned above, both Cdc28/Cdk1-dependent phosphorylation and Ptc1-dependent dephosphorylation of Vac17 are required for the proper formation of the transport complex with Myo2. Interestingly, in *ptc1*Δ cells, the levels of Mmr1, Inp2, and Vac17 are significantly decreased [16]. Moreover, Ptc1 is required for proper localization of Myo2 and Myo4. These results imply that Ptc1 broadly regulates the transport of different organelles carried by class V myosins and thus is an interesting candidate for the master coordinator of organelle inheritance in yeast. Another regulatory factor in Myo2-dependent organelle transport may be the competition for binding sites on Myo2 for adaptor proteins. Myo2 attaches to vesicles and adaptor proteins in its C-terminal globular tail domain, which can be further divided into subdomains I and II. The binding motif of Vac17 is located in subdomain I, whereas secretory vesicles bind to subdomain II [8,37]. The two attachment motifs lie on opposite sides of the globular tail surface and are, therefore, simultaneously exposed to the binding partners. In contrast, the attachment site for Inp2 partially overlaps with the motif for secretory vesicle binding [8]. Therefore, peroxisomes may compete with secretory vesicles, but not vacuoles, for Myo2 attachment. Why the binding motif for Inp2 overlaps with the binding site for secretory vesicles, but not for Vac17, is an intriguing question. Are there any disadvantages if vacuoles spatially compete with peroxisomes for Myo2 attachment? How such partial overlap or converse and distinct placement of cargo-binding sites on the Myo2 globular tail is related to the temporal coordination of organelle transport would be of interest for future research. The regulation of cargo detachment from Myo2 is also differentially regulated in some organelles, despite the overall scheme for unloading being shared by vacuoles and mitochondria. Vacuoles and mitochondria are released from Myo2 through the ubiquitination-dependent proteolysis of adaptor proteins. In contrast, to the best of our knowledge, there are no reports that the adaptor proteins for the Golgi apparatus, Ypt11 and Ypt31/32, are degraded by the proteasome after inheritance. Unlike Vac17 and Mmr1, Ypt11, Ypt31, and Ypt32 all have relatively long lifetimes; the half-lives of Ypt11, Ypt31, and Ypt32 are 14.1 h, 9.7 h, and 10.3 h, respectively [31]. This difference may be due to the involvement of these organelles in cytokinesis progression. During cytokinesis, the Golgi apparatus relocates to the bud neck and generates secretory vesicles containing materials required for septum construction. Therefore, the Golgi apparatus should maintain or form another round of interactions with Myo2 to be carried toward the bud neck during cytokinesis. In contrast, vacuoles and mitochondria are not directly involved in cytokinesis progression, and therefore, their unloading from myosin seems to be beneficial in avoiding the spatial hindrance of septum formation at the bud neck.

Herein, we discuss whether organelle unloading from myosin by ubiquitination-dependent proteolysis of adaptor proteins can be applied more broadly, e.g., to organelle dynamics in mammalian cells. In mammalian cells, mitochondrial location is controlled by microtubules and actin filaments [38,39]. Mammalian cells proliferate by cell division, which is characterized by the partitioning of a pre-existing space, and therefore, they do not require the formation of a novel space. Symmetrical segregation of mitochondria during mammalian cell division requires the mitochondrial myosin Myo19 [40]. However, it has also been reported that mitochondrial segregation during mammalian cell division is passive and does not require mitochondrial transport on the cytoskeleton [41]. In this passive mode of mitochondrial segregation, the mitochondria dissociate from the cytoskeleton before the onset of cell division. Interestingly, prior to passive mitochondrial segregation during mammalian cell division, mitochondrial motor proteins dissociate from the mitochondria, leading to the release of the mitochondria from microtubules [41]. This motor shedding requires the action of protein kinases CDK1 and Aurora A. It would be particularly interesting to determine whether the targeted degradation of mitochondrial motor proteins and/or adaptor proteins is involved in the release of mitochondria from the cytoskeleton for passive segregation during mammalian cell division.

The intracellular transport of melanosomes is another interesting model for studying directed organelle transport. The transport and correct positioning of melanosomes in mammalian cells depend on the cytoskeleton. Melanosomes are first directionally transported by the motor protein kinesin on microtubules, followed by switching over to actin filaments and then being carried by myosin toward the cell surface for secretion [42]. It is natural to consider that such elaborate transport processes should be highly spatially and temporally coordinated. Therefore, it would be interesting for future research to investigate whether ubiquitin-mediated degradation of adaptor proteins is involved in directed melanosome movement.

In *dma1*Δ *dma2*Δ cells, the mitochondria are stacked at the bud tip and neck (Figure 2). Electron microscopy has revealed that these stacked mitochondria are expanded or abnormally deformed [12]. Mmr1, in addition to connecting the mitochondria and Myo2, has been reported to play a role in anchoring transported mitochondria to the bud cortex and facilitating mitochondrial fusion [43]. Failure of Mmr1 degradation in *dma1*Δ *dma2*Δ cells may lead to abnormally accelerated mitochondrial fusion, which in turn results in mitochondrial deformation. It is generally thought that mitochondrial fusion contributes to elevated respiratory activity [44], which is in agreement with the mitochondria in *dma1*Δ *dma2*Δ cells. It would also be intriguing to determine whether the E3 ubiquitin ligases Dma1 and Dma2 indirectly regulate mitochondrial fusion through the ubiquitination and subsequent degradation of Mmr1. The mitophagic activity in *dma1*Δ *dma2*Δ cells may be another interesting topic to be examined in future research. Damaged mitochondria are actively eliminated via mitochondria-selective autophagy, also known as mitophagy. Elevated ROS production may cause mitochondrial damage and invoke mitophagy in *dma1*Δ *dma2*Δ cells. The measurement of mitophagic activity in *dma1*Δ *dma2*Δ cells and investigation of the effects of the deletion of genes required for mitophagy in *dma1*Δ *dma2*Δ cells would be interesting approaches to examine this possibility.

## Abbreviations

ER	endoplasmic reticulum
PEST	proline, glutamic acid, serine, threonine
RING	Really interesting new gene
HOPS	homotypic fusion and vacuole protein sorting
ROS	reactive oxygen species
DIC	differential interference contrast
